# Supplementary material for: Combined targeted and epigenetic-based therapy enhances antitumor immunity by stabilizing GATA6-dependent MHCI expression in pancreatic ductal adenocarcinoma
Source: Nat Commun. 2026 Feb 6;17:1476. doi: 10.1038/s41467-026-69013-y (PMC12886960; doi:10.1038/s41467-026-69013-y)
Supplement: Supplementary file 1 — Supplementary Information [file 41467_2026_69013_MOESM1_ESM.pdf]

Supplementary figure 1

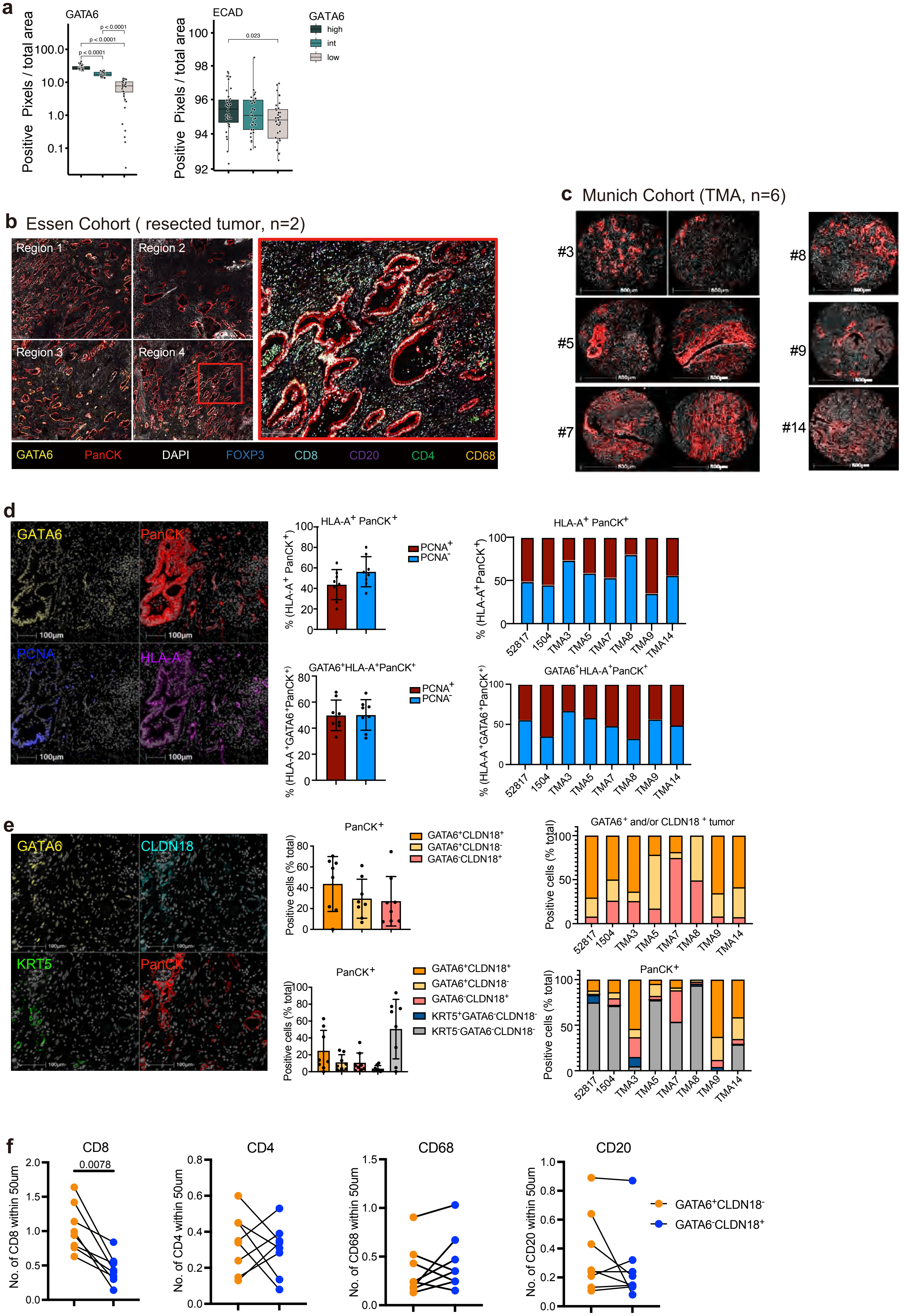

Supplementary Fig. 1: GATA6 expression associates with immunoreactive stroma in human PDAC

(a) GATA6 and E-cadherin (ECAD) were stained by IHC in resections (n=143 biological replicates) and quantified by QuPath bioimage analysis software. Patients were divided into $GATA6^{high}$ (hi), intermediate (int) and low (lo) expression groups based on the GATA6 expression in epithelium. Expression levels of GATA6 and E-cadherin in each group are shown. Statistical significance was calculated by two-sided Wilcoxon rank-sum tests. **(b, c)** Hi-plex immunofluorescent spatial imaging with Phenocycler on 8 human primary treatment-naive PDAC tissues. Among these, 2 samples were **(b)** resected PDAC tissues, while the remaining 6 cases were **(c)** tissue microarray (TMA). **(b)** 4 regions with heterogeneous GATA6 tumor expression were selected for imaging and analysis for resected PDAC tissues, while **(c)** whole tissue cores were included for analyses for TMA tissues. **(d)** Co-expression of GATA6, HLA-A, PCNA in $PanCK^+$ tumor cells in 8 PDAC tissues. Quantified by HALO software. Bar unit: µm. Box plots show the median (centre line), 25th–75th percentiles (bounds of box), and whiskers extending to the minima and maxima values. Individual data points represent independent patients (n = 8). Statistical significance was calculated with unpaired two-tailed Mann–Whitney test. **(e)** Co-expression of GATA6, CLDN18, KRT5 in $PanCK^+$ tumor cells in 8 PDAC tissues. Quantified by HALO software. Bar unit: µm. Statistical significance was calculated with One-way ANOVA, Kruskal–Wallis test. **(f)** Computational spatial analysis of CD8, CD4, CD68 and CD20 with $GATA6^+CLDN18^-$ tumor cells and $GATA6^-CLDN18^+$ counterparts. Statistical significance was calculated with two-tailed Wilcoxon matched-pairs signed rank test.

Supplementary figure 2

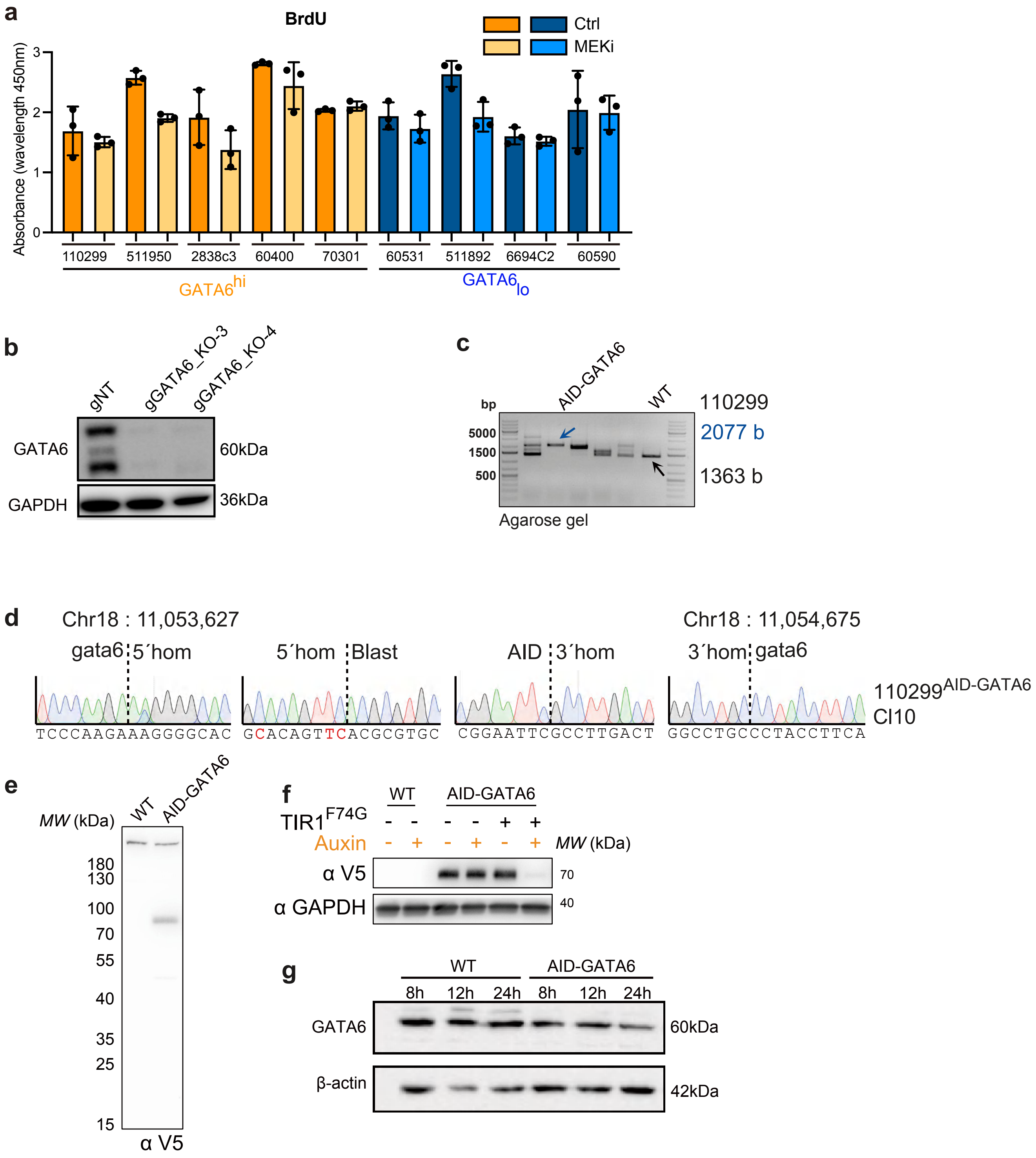

Supplementary Fig. 2: Proliferation profiles of murine PDAC cell lines treated with or without MEKi and the GATA6 modulation in GATA6^{high} murine PDAC cell lines

(a) BrdU assay showing the proliferation level of murine PDAC cell lines with or without MEKi (n = 3 biological replicates per cell line). Individual dots represent independent biological replicates. Mean ± SD is shown. Statistical significance was calculated by One-way ANOVA, Kruskal–Wallis test. **(b)** Western blot showing GATA6 expression of 2838c3 cell lines with GATA6 knockout (gGATA6_KO-3 and gGATA6_KO-4) and negative control (gNT), GAPDH used as a loading control (n = 1 independent sample for each group). **(c)** Agarose gel of genomic PCR from 110299$^{AID\text{-}GATA6}$ knock-in clones. Cl10 (clone 10), homozygous clone and wild type (WT) cells are labelled. **(d)** Sanger sequencing electropherogram of the genomic locus of 110299$^{AID\text{-}GATA6}$ Cl10. 5'hom, 5' homology arm; 3'hom, 3' homology arm; AID, Auxin-inducible degron; Blast, Blasticidin resistance gene. Genomic locations are indicated. The PAM site of the sgRNAs that were mutated using the HDR is shown in red. **(e)** Full-membrane immunoblot of V5-tag in 110299^{WT} (naïve) and 110299$^{AID\text{-}GATA6}$ Cl10 cells. **(f)** Immunoblot of V5-tag. 110299^{WT} and 110299$^{AID\text{-}GATA6}$ Cl10 cells with or without TIR1^{F74G} expression were treated with 1 μM 5-Ph-IAA for 8 hr. GAPDH is used as a loading control. **(g)** Western blot showing GATA6 protein expression in 110299^{WT} and 110299$^{AID\text{-}GATA6}$ cells after 8, 12 and 24h treatment with 1 μM 5-Ph-IAA. WT: 110299^{WT}; AID-GATA6: 110299$^{AID\text{-}GATA6}$, β-actin used as a loading control (n = 1 independent sample for each group).

Supplementary figure 3

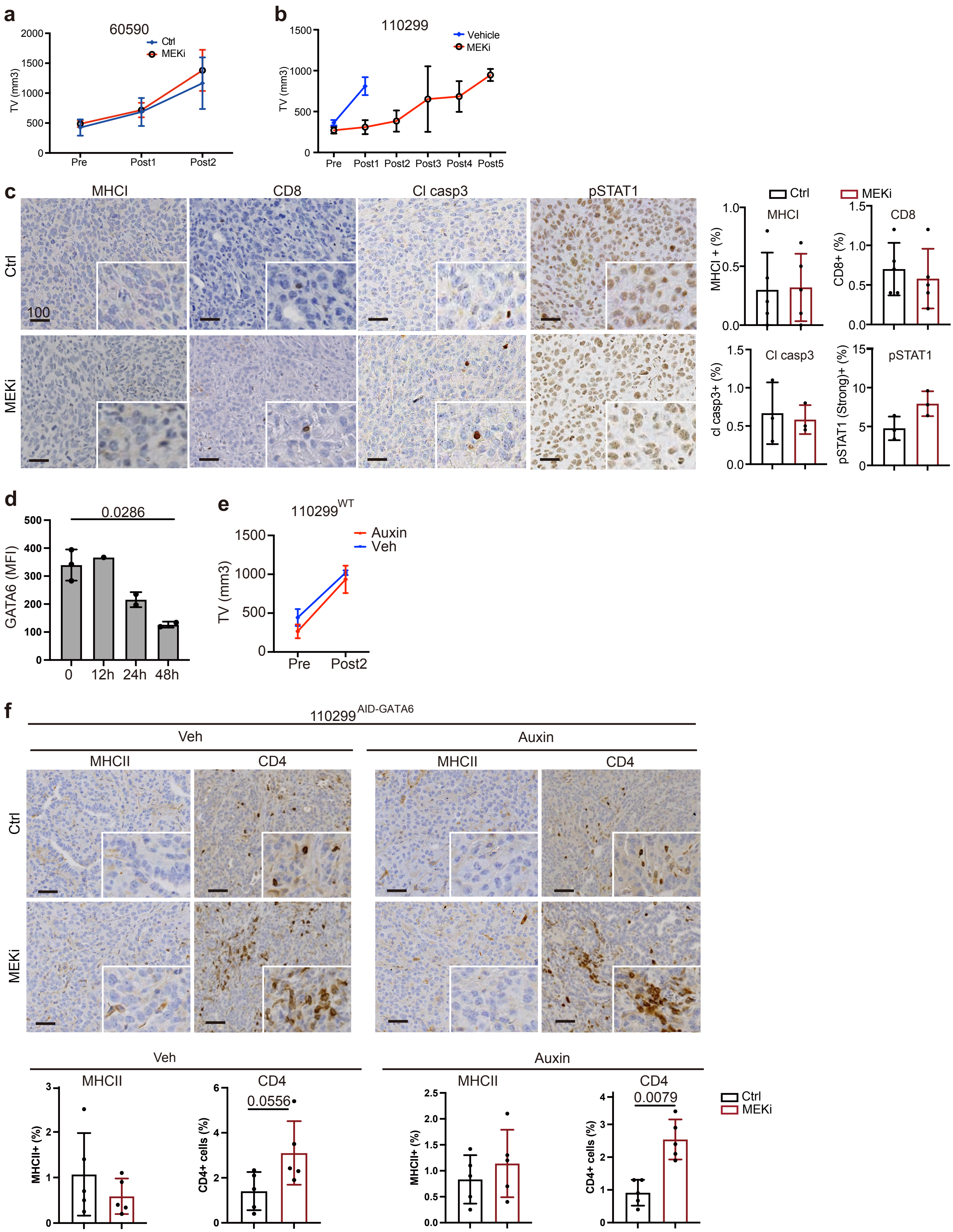

Supplementary Fig. 3: GATA6 is essential for MEKi-induced tumor control and anti-tumor cytotoxicity *in vivo*

Tumor growth curves of orthotopic tumor model of **(a)** $GATA6_{low}$ 60590 (n = 4 independent mice) and **(b)** $GATA6^{high}$ 110299 cells (n = 5 independent mice) treated with or without MEKi. TV: Tumor volume. **(c)** IHC staining of MHCI, CD8, Cl casp3 and pSTAT1 in orthotopic tumor model of $GATA6_{low}$ 60590 treated with or without MEKi (n = 5 independent mice for MHCI, CD8 and n = 3 independent mice for Cl casp3, pSTAT1). Data are presented as mean values ± SD. Individual data points represent independent mice. Statistical significance was calculated by unpaired two-tailed Mann–Whitney test. **(d)** Flow cytometric analysis of GATA6 expression in orthotopic tumor models of $110299^{AID\text{-}GATA6}$ treated with auxin for different time points (n = 3 independent mice for 0h, n = 1 for 12h and n = 2 independent mice for 48h). Data are presented as mean values ± SD. Individual data points represent independent mice. Statistical significance was calculated by One-way ANOVA, Kruskal–Wallis test. **(e)** Tumor growth curve of orthotopic tumor models of 110299^{WT} with or without MEKi (n = 3 independent mice for each group). Data are presented as mean values ± SD. Individual data points represent independent mice. **(f)** IHC staining of MHCI and CD4 in orthotopic tumor models of $110299^{AID\text{-}GATA6}$ treated with or without MEKi (n = 5 independent mice for each group). Data are presented as mean values ± SD. Individual data points represent independent mice. Statistical significance was calculated by unpaired two-tailed Mann–Whitney test. Ctrl: vehicle control for MEKi; Veh: vehicle for auxin; Scale bars: µm.

Supplementary figure 4

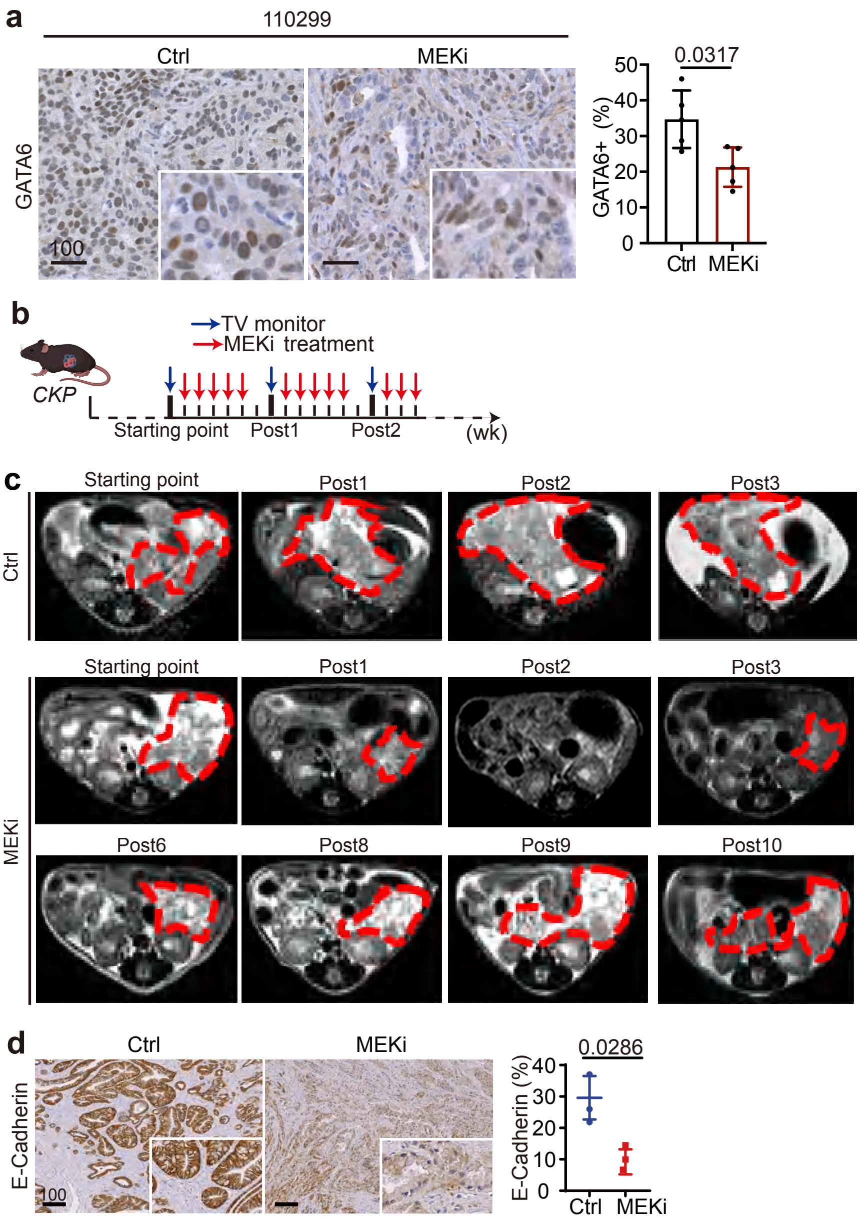

Supplementary Fig. 4: HDAC inhibitors restored GATA6 expression and promoted MHCI expression

(a) IHC staining of GATA6 of orthotopic tumors in the GATA6^{high} 110299 upon MEKi treatment. (n = 5 independent mice for each group). Data are presented as mean values ± SD. Individual data points represent independent mice. Statistical significance was calculated by unpaired two-tailed Mann–Whitney test. Scale bar: µm. **(b)** Timeline for treatment of *CKP* mice with vehicle control (Ctrl) or MEKi (trametinib). Red arrows indicate the treatment schedule, in which mice were treated once daily for 5 consecutive days, followed by 2 days off treatment. Blue arrows indicate the schedule of tumor volume (TV) monitoring by MRI imaging. Created with BioRender.com. **(c)** Representative MR images of a Ctrl and MEKi treated *CKP* mouse at therapy starting point as well as post-treatment time points. Red-doted lines illustrate the tumor location and size. **(d)** IHC staining of epithelial marker E-Cadherin in *CKP* tumors. High-magnification images show the intratumoral heterogeneity of E-Cadherin expression in MEKi treated tumor. The percentage of E-Cadherin positive cells in the whole tumorous tissues as quantified by Definiens software. (n = 4 independent mice for each group). Data are presented as mean values ± SD. Individual data points represent independent mice. Statistical significance was calculated by unpaired two-tailed Mann–Whitney test. Scale bar: µm.

Supplementary figure 5

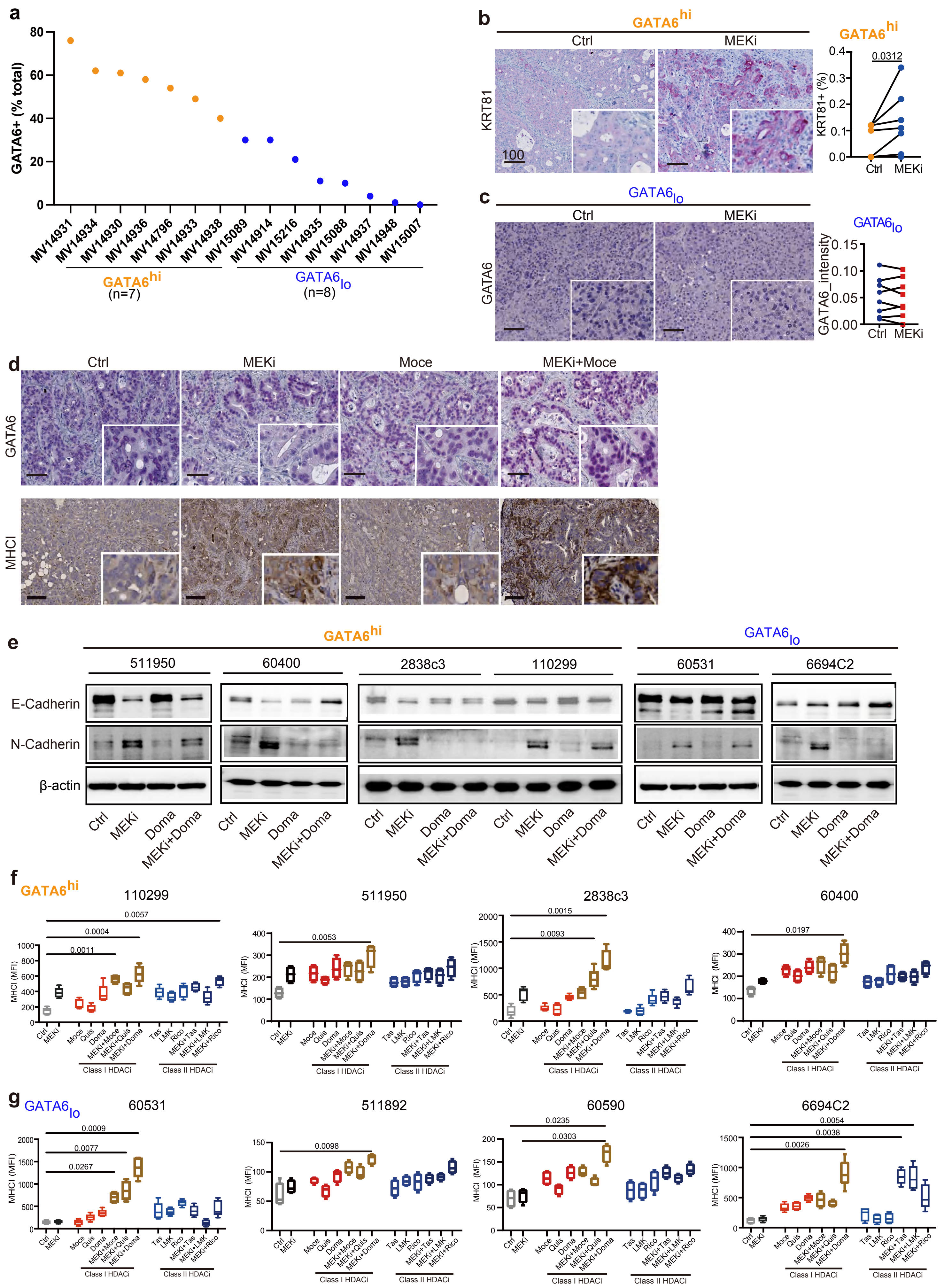

Supplementary figure 5-2

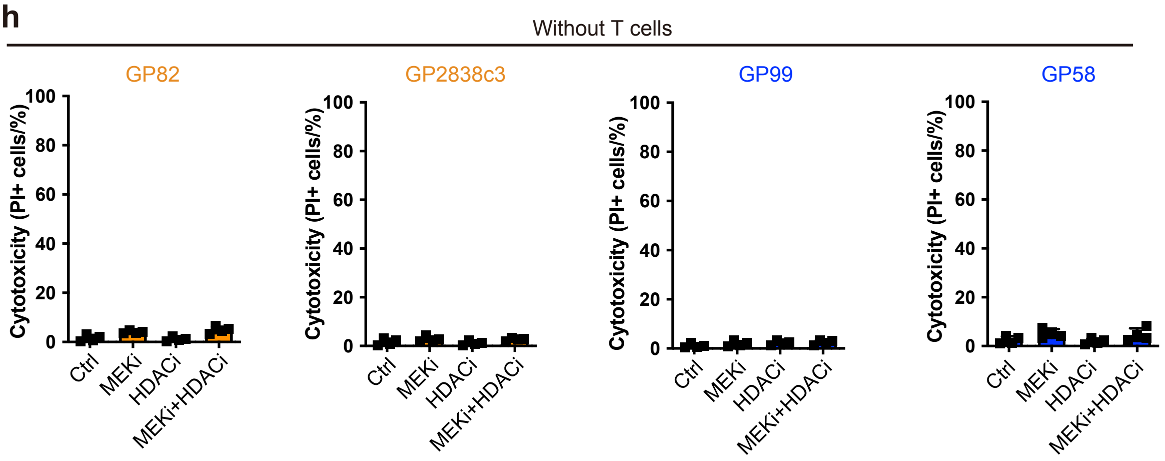

Supplementary Fig. 5: HDAC inhibitors restored GATA6 expression and promoted MHCI expression

(a) IHC staining of GATA6 expression was performed on all 15 PDX xenograft tumors. GATA6 expression (positive cells, % of total cells) of tumors was quantified by Definiens software. Median of GATA6 expression was used as cutoff to dichotomize $GATA6^{high}$ ($GATA6^{hi}$) and $GATA6_{low}$ ($GATA6_{lo}$) groups (n = 7 independent mice for $GATA6^{hi}$ and n = 8 independent mice $GATA6_{lo}$). **(b)** IHC staining of basal-like marker KRT81 in $GATA6^{high}$ PDX treated with or without MEKi. (n=7 independent mice). The percentage of $KRT81^{+}$ cells in the tumorous tissues as quantified by Definiens software. Statistical significance was calculated by Wilcoxon matched-pairs signed rank test. Scale bar: µm. **(c)** IHC staining of GATA6 expression in $GATA6_{low}$ PDX tumors. GATA6 expression (positive cells, % of total cells) of the tumorous tissues as quantified by Definiens software (n=8 independent mice). Statistical significance was calculated by Wilcoxon matched-pairs signed rank test. **(d)** IHC staining of GATA6 and MHCI in PDX treated with or without MEKi and/or mocetinostat (Moce). Ctrl: vehicle control; MEKi: trametinib; Moce: mocetinostat. Scale bar: µm. **(e)** Western blot analysis of the expression levels of EMT-related markers (E-cadherin and N-cadherin) in murine PDAC cells upon 4 treatment groups. β-actin used as a loading control (n = 1 independent sample). **(f)** $GATA6^{high}$ and **(g)** $GATA6_{low}$ murine PDAC cell lines were treated with or without MEKi and/or class I HDACi: mocetinostat (moce), quisinostat (quis), domatinostat (doma); class II HDACi: tasquinimod (tasq), LMK235 (LMK), ricolinostat (rico), for 72h and the cell surface MHCI (H-2Db) expression was assessed by flow cytometry (n = 4 independent experiments for the cell lines 511950, 60400, 511892, 60590,

and n = 5 independent experiments for the cell lines 110299, 2838c3, 60531, 6694C2). Data are presented as mean values ± SD. Statistical significance was calculated by One-way ANOVA, Kruskal–Wallis test. MFI: mean fluorescence intensity. **(h)** Cytotoxicity level (PI^+ %) of GP cells without LCMV-gp33-reactive T cells treated with or without MEKi and/or HDACi (n = 4 independent experiments). Data are presented as mean values ± SD. Statistical significance was calculated by One-way ANOVA, Kruskal–Wallis test. Ctrl: vehicle control; MEKi: trametinib; HDACi: domatinostat. Scale bar: µm.

Supplementary figure 6

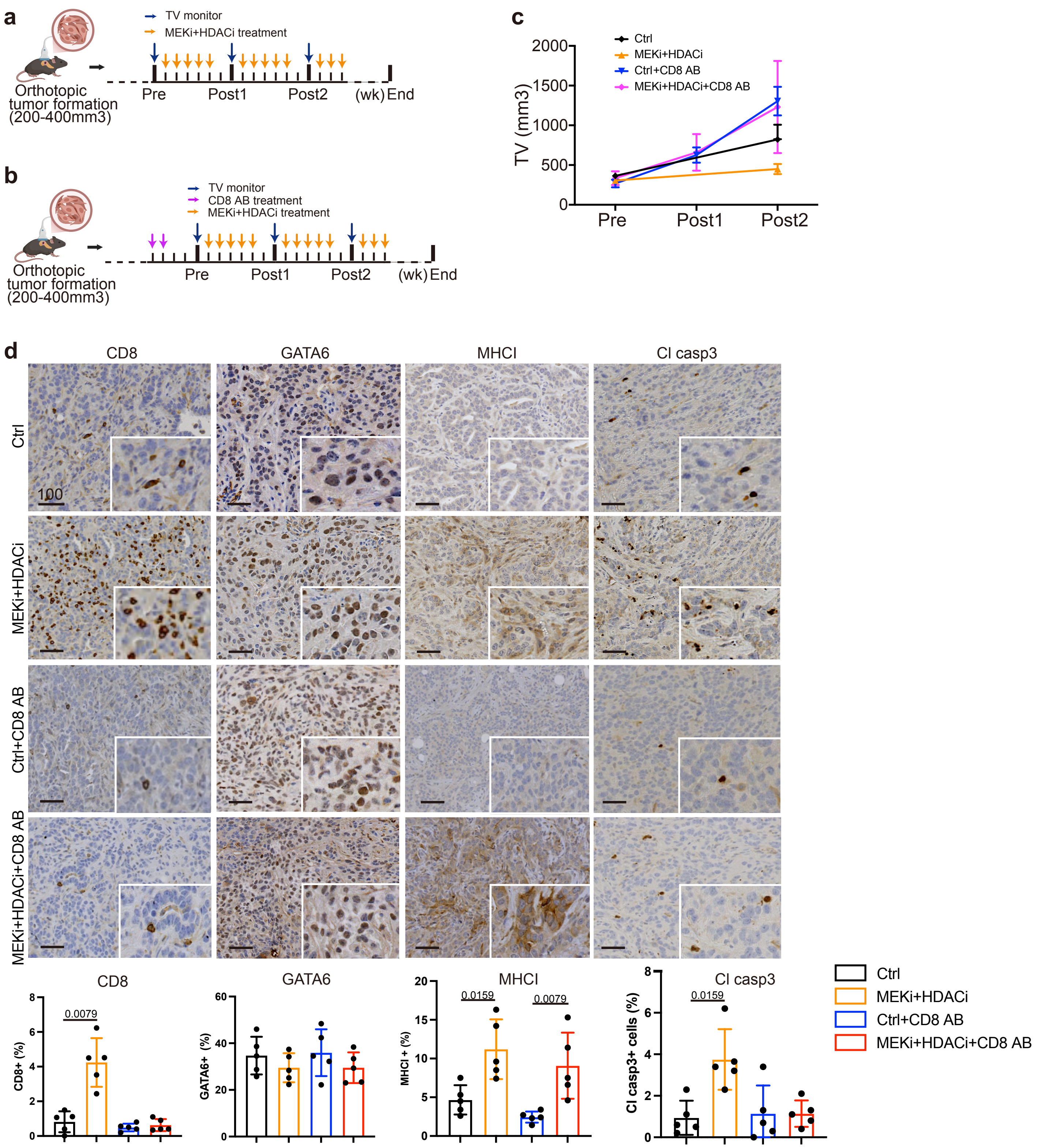

Supplementary Fig. 6: MEKi and HDACi combination treatment enhances $CD8^+$ T cell-mediated tumor suppression

(a) Treatment schedule of the combination therapy of MEKi and HDACi in orthotopic mouse model derived from the $GATA6^{high}$ cell line 110299. Created with BioRender.com. **(b)** Treatment schedule of the combination therapy of MEKi and HDACi in orthotopic mouse model derived from the $GATA6^{high}$ cell line 110299 with prior CD8 depletion. Created with BioRender.com. **(c)** Tumor growth curves of orthotopic mouse model derived from the $GATA6^{high}$ cell line 110299 upon combination therapy of MEKi and HDACi with or without CD8 depletion (n = 5 for Veh and Veh+CD8 Ab, n = 4 for MEKi+HDACi and MEKi+HDACi+CD8 AB). TV: Tumor volume. **(d)** IHC staining of CD8, GATA6, MHCI and Cl casp3 in orthotopic tumor model of the $GATA6^{high}$ cell line 110299 upon combination therapy of MEKi and HDACi with or without CD8 depletion. The lower panel shows the percentage of respective positive cells in the whole tumorous tissues as quantified by HALO software. (n = 5 independent mice for each group). Data are presented as mean values ± SD. Individual data points represent independent mice. Statistical significance was calculated by One-way ANOVA, Kruskal–Wallis test. Scale bar: µm. Ctrl: vehicle control; MEKi: refametinib; HDACi: domatinostat; CD8 AB: CD8 antibody.

Supplementary figure 7

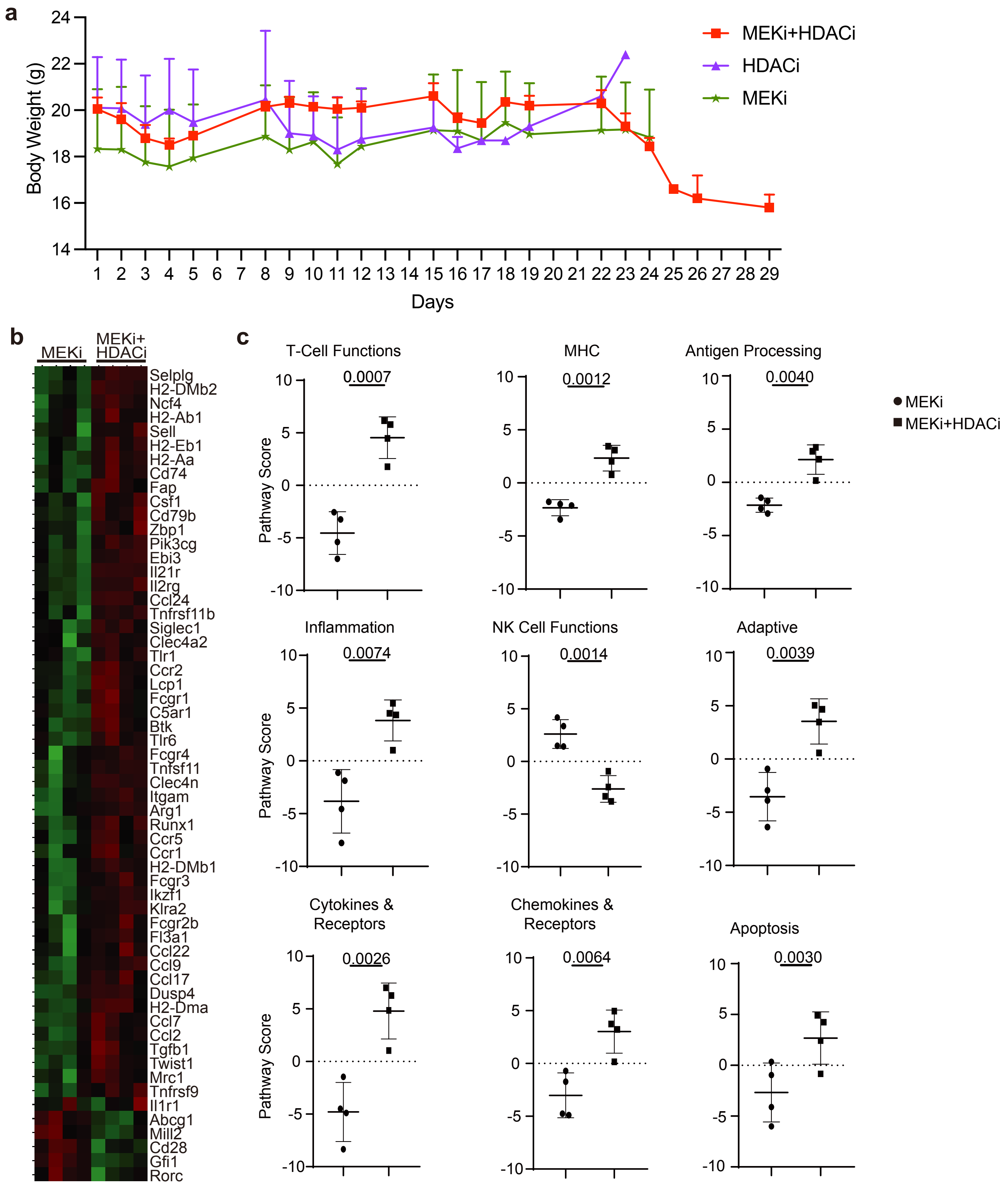

Supplementary Fig. 7: Combination of MEKi and HDACi prolongs survival of *CKP* mice with restored GATA6 and MHCI expression

(a) Body weight curves of *CKP* mice across different treatment groups (n = 2 for MEKi+ HDACi, n = 4 for HDACi, n = 3 for MEKi). **(b)** Heatmap of the differential immune-related genes expression analysis by NanoString PanCancer Immune Profiling Panel, MEKi vs MEKi+HDACi (n = 4 independent mice for each group) with adjusted p-value<0·05, Benjamini-Yekutieli false discovery rate (FDR) < 0.1. Red indicates high log fold-change, green indicates low log fold-change. **(c)** Pathways with significant difference between MEKi vs MEKi+HDACi (n = 4 independent mice for each group). Data are presented as mean values ± SD. Individual data points represent independent mice. Statistical significance was assessed with unpaired two-tailed Mann–Whitney test. MEKi: trametinib; HDACi: domatinostat; MEKi+HDACi: trametinib + domatinostat.

Supplementary Tables

Supplementary Table 1 : Clinicopathological information of 8 therapy-naive human PDAC samples for highly multiplex spatial imaging with Phenocycler

Cases	Age	Sex	Grading	pT	pN
TMA_3	85	F	3 or 4	3	2
TMA_5	62	M	3		
TMA_7	41	M	2	3	1
TMA_8	83	F	3	3	1
TMA_9	67	M	2	3	0
TMA_14	71	M	2		
Resected_1	70	F	3	3	1
Resected_2	65	M	3	3	0

Supplementary Table 2: Sequential multiplexed immunofluorescence staining protocol

Antigen	Primary antibody				TSA fluorophore	
	**Clone**	**Manufacturer**	**Catalogue, lot. number**	**Dilution**	**Fluorophore**	**Dilution**
**Fig. 5f**						
MHCI	27-11-13	Abcam	ab25244, lot: 1005013-1;	1:100	Opal480	1:200
GATA6	Polyclonal	Abcam	ab175349, lot: GR3447918-1	1:500	Opal690	1:200
PanCK	PCK-26	Abcam	ab6401, lot: 1036440-2;	1:100	Opal780	1:200
**Fig. 7a**						
MHCI	27-11-13	Abcam	ab25244, lot: 1005013-1;	1:100	Opal480	1:200
cl. casp3	5A1E	Cell signaling	9664L, lot: 22;	1:100	Opal570	1:200
GzmB	Polyclonal	Abcam	ab4059, lot: GR3199533-2	1:200	Opal690	1:200
PanCK	PCK-26	Abcam	ab6401, lot: 1036440-2;	1:100	Opal780	1:100

Supplementary Table 3: IHC antibodies

Antigen	Clone	Manufacturer	Catalogue, lot. number	Dilution
GATA6	Polyclonal	Abcam	ab175349, lot: GR3447918-1	1:200
PanCK	PCK-26	Abcam	ab6401, lot: 1036440-2;	1:100
Cl. caspase 3	5A1E	Cell Signaling	9664L, lot: 22;	1:100
MHC II	NIMR-4	Abcam	ab25333; lot: GR3203074-1	1:100
CD4	EPR19514	Abcam	ab183685, lot: GR3245678-1	1:100
CD8	EPR20305	Abcam	ab209775, lot: 1014501-40	1:100
GzmB	Polyclonal	Abcam	ab4059, lot: GR3199533-2	1:100
MHC I (H-2Db)	R1-21.2	Abcam	ab281902, lot: 1022142-4	1:100
PD1	EPR20665	Abcam	ab214421, lot: 1007880-1	1:100
pSTAT1	EPR3146	Abcam	ab109461; lot: 1000296-47	1:1000
E-Cadherin	24E10	Cell Signaling	3195S; lot: 15	1:200
