# Supplementary material for: Combined targeted and epigenetic-based therapy enhances antitumor immunity by stabilizing GATA6-dependent MHCI expression in pancreatic ductal adenocarcinoma
Source: Nat Commun. 2026 Feb 6;17:1476. doi: 10.1038/s41467-026-69013-y (PMC12886960; doi:10.1038/s41467-026-69013-y)
Supplement: Supplementary file 2 — Description of Additional Supplementary Files [file 41467_2026_69013_MOESM2_ESM.pdf]

Description of Additional Supplementary Files

Supplementary Dataset 1: Source Data for Figure 1a and Figure S1a.

Supplementary Dataset 2: GSEA in Maurer et al dataset (GSE93326).

Supplementary Dataset 3: GSEA in Chen et al dataset (GSE212966).

Supplementary Dataset 4: GSEA in Godfrey et al dataset (GSE146348).

Supplementary Dataset 5: GSVA of NanoString PanCancer Immune Profiling.

Supplementary Dataset 6: (a) CODEX and (b) Phenocycler/FUSION antibody information.
