# Supplementary material for: Combined targeted and epigenetic-based therapy enhances antitumor immunity by stabilizing GATA6-dependent MHCI expression in pancreatic ductal adenocarcinoma
Source: Nat Commun. 2026 Feb 6;17:1476. doi: 10.1038/s41467-026-69013-y (PMC12886960; doi:10.1038/s41467-026-69013-y)
Supplement: Supplementary file 9 — Reporting Summary [file 41467_2026_69013_MOESM9_ESM.pdf]

nature portfolio

Corresponding author(s): Jens T Siveke

Last updated by author(s): Sep 23, 2025

Reporting Summary

Nature Portfolio wishes to improve the reproducibility of the work that we publish. This form provides structure for consistency and transparency in reporting. For further information on Nature Portfolio policies, see our Editorial Policies and the Editorial Policy Checklist.

Statistics

For all statistical analyses, confirm that the following items are present in the figure legend, table legend, main text, or Methods section.

n/a	Confirmed	
☐	☒	The exact sample size (*n*) for each experimental group/condition, given as a discrete number and unit of measurement
☐	☒	A statement on whether measurements were taken from distinct samples or whether the same sample was measured repeatedly
☐	☒	The statistical test(s) used AND whether they are one- or two-sided<br>*Only common tests should be described solely by name; describe more complex techniques in the Methods section.*
☒	☐	A description of all covariates tested
☐	☒	A description of any assumptions or corrections, such as tests of normality and adjustment for multiple comparisons
☐	☒	A full description of the statistical parameters including central tendency (e.g. means) or other basic estimates (e.g. regression coefficient) AND variation (e.g. standard deviation) or associated estimates of uncertainty (e.g. confidence intervals)
☐	☒	For null hypothesis testing, the test statistic (e.g. *F*, *t*, *r*) with confidence intervals, effect sizes, degrees of freedom and *P* value noted<br>*Give P values as exact values whenever suitable.*
☒	☐	For Bayesian analysis, information on the choice of priors and Markov chain Monte Carlo settings
☒	☐	For hierarchical and complex designs, identification of the appropriate level for tests and full reporting of outcomes
☒	☐	Estimates of effect sizes (e.g. Cohen's *d*, Pearson's *r*), indicating how they were calculated

Our web collection on statistics for biologists contains articles on many of the points above.

Software and code

Policy information about availability of computer code

Data collection	Microscope: Axio Scanner Z.1 (Zeiss), BZ-X710 inverted fluorescence (Keyence), Flow cytometry: FACSCelesta (BD); mRNA profiling: nanoString Technologies
Data analysis	Microscopic images: Definiens Tissue Studio (ver 2.6), ZEISS ZEN (ver 3.1) Imaging Software. HALO (ver 3.2) for spatial image analysis; Flow cytometry: FACS Diva v.5, FlowJo ver 7.5.5; mRNA profiling: nSolver (ver 4.0); Transcriptional profiling of dataset from Maurer et al (GSE93326), Chen et al (GSE212966): Hallmarks (h.all.v7.5.1), KEGG (2.cp.kegg.v7.5.1), and GO (c5.go.v7.5.1), R software (ver 4.1.3) package 'DESeq2', 'Seurat', 'CellChat', 'ClusterProfiler'; Other statistical analyses: GraphPad Prism (ver 8.0).

For manuscripts utilizing custom algorithms or software that are central to the research but not yet described in published literature, software must be made available to editors and reviewers. We strongly encourage code deposition in a community repository (e.g. GitHub). See the Nature Portfolio guidelines for submitting code & software for further information.

Data

Policy information about availability of data

All manuscripts must include a data availability statement. This statement should provide the following information, where applicable:

- Accession codes, unique identifiers, or web links for publicly available datasets
- A description of any restrictions on data availability
- For clinical datasets or third party data, please ensure that the statement adheres to our policy

The data used to analyze the transcriptomic profiling of GATA6 expression in PDAC is available in Gene Expression Omnibus (GEO) database under accession code GSE93326, GSE212966. The RNA sequencing data is available in the GEO database under accession code GEO: GSE146348.

Research involving human participants, their data, or biological material

Policy information about studies with human participants or human data. See also policy information about sex, gender (identity/presentation), and sexual orientation and race, ethnicity and racism.

Reporting on sex and gender	IHC staining of GATA6 and stroma/immune markers was performed in patient samples obtained from the UHN Biospecimens Program. The information of the patient cohort including sex of subjects has been published in Grunwald BT et al, Cell, 2021. The two resected primary PDAC cases (one female and one male sex) that were analyzed by the highly multiplex spatial imaging with CODEX were part of the 'Essen' cohort, a retrospective cohort consisting of patients that had undergone pancreatic resection with a final histopathologic diagnosis of human PDAC between March 2006 and February 2016. The tissue microarray analyzed by the highly multiplex spatial imaging with Phenocycler was compiled at the Institute of Pathology, Technical University of Munich, consisting of primary resected PDAC, resected between April 2008 and May 2020. Four out of six tissues were obtained from male sex patients and two of six from female sex patients.
Reporting on race, ethnicity, or other socially relevant groupings	No data about race, ethnicity, or other socially relevant groupings was collected for this study.
Population characteristics	IHC staining of GATA6 and stroma/immune markers was performed in patient samples obtained from the UHN Biospecimens Program. The information of the patient cohort including sex of subjects has been published in Grunwald BT et al, Cell, 2021. The clinicopathological data of the patients analyzed by the highly multiplex spatial imaging with CODEX/ Phenocycler are shown in supplementary table 5.
Recruitment	Patient samples used for GATA6 and stroma/immune marker staining were mostly accrued at Princess Margaret Cancer Centre at the University Health Network (Toronto, Canada). Since the resected primary PDAC samples analyzed by the highly multiplex spatial imaging with CODEX were part of a retrospective study for PDAC patients we did not participate in the patients recruitment process. The tissue microarray analyzed by the highly multiplex spatial imaging with Phenocycler was compiled at the Institute of Pathology, Technical University of Munich, consisting of primary resected PDAC, from patients mostly accrued there and resected between April 2008 and May 2020.
Ethics oversight	UHN Biospecimens Program: University Health Network Research Ethics Board (Nos. 03-0049, 08-0767, 15-9596, 17-6106, 16-5380). Resected primary PDAC samples analyzed by the highly multiplex spatial imaging with CODEX- 'Essen' cohort: according to the recommendations of the local ethics committee of the Medical Faculty of the University of Duisburg-Essen, approval no: 17-7340-BO. TMA analyzed by the highly multiplex spatial imaging with Phenocycler: Technical University of Munich, ethical vote: 403/17 S

Note that full information on the approval of the study protocol must also be provided in the manuscript.

Field-specific reporting

Please select the one below that is the best fit for your research. If you are not sure, read the appropriate sections before making your selection.

☒ Life sciences ☐ Behavioural & social sciences ☐ Ecological, evolutionary & environmental sciences

For a reference copy of the document with all sections, see nature.com/documents/nr-reporting-summary-flat.pdf

Life sciences study design

All studies must disclose on these points even when the disclosure is negative.

Sample size	IHC staining of GATA6 and stroma/immune markers was performed in patient samples obtained from the UHN Biospecimens Program. The study includes 143 resectable tumor specimens from treatment-naïve patients with stage I/II PDAC. 8 tissues in total were included in the highly multiplex spatial imaging analysis with CODEX/ Phenocycler.<br>For in vivo experiment, PDXs from 15 PDAC patients were transplanted subcutaneously into athymic mice (n= 7 GATA6 high vs 8 GATA6 low). 2-4 mice were used per patient for drug treatment. For the rest of the mouse studies at least 6 mice were included per group. The sample size was calculated by power analysis.

Data exclusions	No data was excluded from the analyses of this study. None of the mice with the appropriate genotype was excluded.
Replication	For human cohorts, IHC stainings were performed on FFPE samples of patients in stage I/II included in the UHN Biospecimens Program (n=143). For CODEX /Phenocycler staining on FFPE human PDAC samples: 4 regions in the same resected tissue and the whole TMAs were analyzed.<br>For mouse studies, IHC staining was performed on all animals included in the experiments (PDXs, n= 7 GATA6 high vs 8 GATA6 low; CKPs, at least 4 mice per treatment group).<br>mIF stainings were performed on all samples of each treatment group of CKP mice (vehicle control, n=4 ; MEKi: n=4; HDACi: n=5; MEKi + HDACi: n= 6, with 7 analyzed areas). All stainings were quantified and analyzed by software. Quantified results were all shown in the manuscript.<br>All in vitro assays were repeated at least 3 times. All attempts at replication were successful.
Randomization	All sample allocation was random in the study.
Blinding	The investigators were blinded to group allocation during data collection and analysis.

Reporting for specific materials, systems and methods

We require information from authors about some types of materials, experimental systems and methods used in many studies. Here, indicate whether each material, system or method listed is relevant to your study. If you are not sure if a list item applies to your research, read the appropriate section before selecting a response.

Materials & experimental systems

n/a	Involved in the study
☐	☒ Antibodies
☐	☒ Eukaryotic cell lines
☐	☐ Palaeontology and archaeology
☐	☒ Animals and other organisms
☐	☐ Clinical data
☐	☐ Dual use research of concern
☐	☐ Plants

Methods

n/a	Involved in the study
☐	☐ ChIP-seq
☐	☒ Flow cytometry
☐	☐ MRI-based neuroimaging

Antibodies

Antibodies used

Immunohistochemistry & mIF
MHCI, Clone: 27-11-13, Manufacturer: Abcam, ab25244, lot: 1005013-1;
GATA6, Clone: Polyclonal, Manufacturer: Abcam, ab175349, lot: GR3447918-1;
PanCK, Clone: PCK-26, Manufacturer: Abcam, ab6401, lot: 1036440-2;
Cl. caspase 3, Clone: 5A1E, Manufacturer: Cell Signaling, 9664L, lot: 22;
MHC II, Clone: NIMR-4, Manufacturer: Abcam, ab25333; lot: GR3203074-1;
CD4, Clone: EPR19514, Manufacturer: Abcam, ab183685, lot: GR3245678-1;
CD8, Clone: EPR20305, Manufacturer: Abcam, ab209775, lot: 1014501-40;
Granzyme B, Clone: Polyclonal, Manufacturer: Abcam, ab4059, lot: GR3199533-2;
MHC I (H-2Db), Clone: R1-21.2, Manufacturer: Abcam, ab281902, lot: 1022142-4
PD1, Clone: EPR20665, Manufacturer: Abcam, ab214421, lot: 1007880-1;
pSTAT1, Clone: EPR3146, Manufacturer: Abcam, ab109461; lot: 1000296-47:
E-Cadherin, Clone: 24E10, Manufacturer: Cell Signaling, 3195S; lot: 15.

Flow cytometry:
H2Db, APC, Clone: KH95, Manufacturer: Biolegend, 111513, lot: B351983;
GzmB, Alexa Fluor 647, Clone: GB11, Manufacturer: Biolegend, 515406, lot: B301362;
TNFa, PE, Clone: MP6-XT22, Manufacturer: Biolegend, 506306, lot: B290019;
PD1, FITC Clone: 29F.1A12, Manufacturer: Biolegend, 135213;
GATA6, PE, Clone: D61E4, Manufacturer: Cell Signaling Technology, 26452, lot: 4.

CODEX:
CD31-BX001, Alexa Fluor 750, Manufacture: Akoya, PN 232172, lot: B347644;
TIGIT-BX002, Atto 550, Manufacture: Akoya, PN 4250061;
CD4-BX003, Cy5, Manufacture: Akoya, PN 4550112;
HLA-A-BX004, Alexa Fluor 750, Manufacture: Akoya, PN 4450046;
Claudin-18-BX005, Atto 550, Manufacturer: Sigma, HPA018446, lot: 000025322;
CD107a-BX006, Cy5, Manufacture: Akoya, PN 232125, lot: B350898;
CD20-BX007, Alexa Fluor 750, Manufacture: Akoya, PN 232175, lot: B382574;
SMA-BX013, Alexa Fluor 750, Manufacture: Akoya, PN 240068, lot: B376070;
CD68-BX015, Cy5, Manufacture: Akoya, PN 232176, lot: B346358;
CD45RO-BX017, Atto 550, Manufacture: Akoya, PN 232188, lot: B328418;
Pan-Cytokeratin-BX019, Alexa Fluor 750, Manufacture: Akoya, PN 232180, lot: B349937;
IFN-γ-BX020, Atto 550, Manufacture: Akoya, PN 240081, lot: B369226;

Podoplanin-BX023, Atto 550, Manufacture: Akoya, PN 232129, lot: B355599;
CD11c-BX024, Cy5, Manufacture: Akoya, PN 232177, lot: B377402;
CD8-BX026, Atto 550, Manufacture: Akoya, PN 232151, lot: 1000000301;
FOXP3-BX031, Alexa Fluor 647, Manufacture: Akoya, PN 4550071;
HLA-DR-BX033, Cy5, Manufacture: Akoya, PN 240017, lot: B347645;
GATA6-BX040, Alexa Fluor 750, Manufacture: R&D, AF1700, lot: KWT0521111;
Granzyme B-BX041, Atto 550, Manufacture: Akoya, PN 240074, lot: B372374;
PD-L1-BX043, Alexa Fluor 647, Manufacture: Akoya, PN 240171, lot: B375169;
CD3e-BX045, Cy5, Manufacture: Akoya, PN 240006, lot: B367598;
PD-1-BX046, Alexa Fluor 647, Manufacture: Akoya, PN 240035, lot: B381164;
Ki67-BX047, Atto 550, Manufacture: Akoya, PN 232179, lot: B350899;
FAP-BX052, Alexa Fluor 750, Manufacture: R&D, AF3715, lot: ZKW0622011;
LAG3-BX055, Alexa Fluor 647, Manufacturer: Akoya, PN: 240077, lot: B388271;
TP63-BX093, Alexa Fluor 647, Manufacture: Akoya, PN 240179, lot: B363038;
Keratin 5-BX101, Alexa Fluor 750, Manufacture: Akoya, PN 240189, lot: B389468;

Phenocycler FUSION:
CD31-BX001, Alexa Fluor 750, Manufacturer: Akoya, PN: 232172, lot: B347644;
CD4-BX003, Alexa Fluor 647, Manufacturer: Akoya, PN: 232174, lot: 0923L470;
HLA-A-BX004, Alexa Fluor 750, Manufacturer: Akoya, PN: 240065, lot: 1000000184;
Claudin-18-BX005, Atto 550, Manufacturer: Sigma, PN: HPA018446, lot: 000025322;
CD107a-BX006, Alexa Fluor 647, Manufacturer: Akoya, PN: 232125, lot: 0324L745;
CD20-BX007, Alexa Fluor 750, Manufacturer: Akoya, PN: 232175, lot: 1000000194;
Has1-BX010, Alexa Fluor 750, Manufacturer: Abcam, ab198846
SMA-BX013, Alexa Fluor 750, Manufacturer: Akoya, PN: 240068, lot: 1000000283;
LRRC15-BX014, Atto 550, Manufacturer: Abcam, ab150376;
CD68-BX015, Alexa Fluor 647, Manufacturer: Akoya, PN: 232176, lot: B346358;
CD66-BX016, Alexa Fluor 647, Manufacturer: Akoya, PN: 240067, lot: B399543;
CD45RO-BX017, Atto 550, Manufacturer: Akoya, PN: 232188, lot: 1023L220;
Pan-Cytokeratin-BX019, Alexa Fluor 750, Manufacturer: Akoya, PN: 232180, lot: 1000000191;
IFN-γ-BX020, Atto 550, Manufacturer: Akoya, PN: 240081, lot: B369226;
CXCR3-BX021, Alexa Fluor 647, Manufacturer: Abcam, PN: ab288437;
Podoplanin-BX023, Atto 550, Manufacturer: Akoya, PN: 232129, lot: B355599;
CD34-BX025, Alexa Fluor 750, Manufacturer: Akoya, PN: 240076 , lot: 0123L495;
CD8-BX026, Atto 550, Manufacturer: Akoya, PN: 232151, lot: B343456;
CD56-BX028, Atto 550, Manufacturer: Akoya, PN: 240186, lot: B398076;
GATA6-BX029, Atto 550, Manufacturer: R&D, AF1700, lot: KWT0521111;
MARCO-BX030, Alexa Fluor 647, Manufacturer: Abcam, PN: ab271060;
HLA-DR-BX033, Alexa Fluor 647, Manufacturer: Akoya, PN: 240017, lot: 1000000096;
CXCR4-BX034, Alexa Fluor 750, Manufacturer: Abcam, PN: ab197203;
PCNA-BX036, Alexa Fluor 647, Manufacturer: Akoya, PN: 240073, lot: B378417;
Granzyme B-BX041, Atto 550, Manufacturer: Akoya, PN: 240074, lot: B372374;
PD-L1-BX043, Alexa Fluor 647, Manufacturer: Akoya, PN: 240171, lot: B375169;
CD3e-BX045, Cy5, Manufacturer: Akoya, PN: 240006, lot: 1000000201;
PD-1-BX046, Alexa Fluor 647, Manufacturer: Akoya, PN: 240035, lot: B381164;
Ki67-BX047, Atto 550, Manufacturer: Akoya, PN: 232179, lot: 1000000102;
Progranulin-BX050, Alexa Fluor 647, Manufacturer: R&D, PN: AF2557;
FAP-BX052, Alexa Fluor 750, Manufacturer: R&D, PN: AF3715, lot: ZKW0622011;
MCT4-BX054, Atto 550, Manufacturer: Abcam, PN: ab244385;
LAG3-BX055, Alexa Fluor 647, Manufacturer: Akoya, PN: 240077, lot: B388271;
TCF-1-BX061, Alexa Fluor 647, Manufacturer: Akoya, PN: 240127, lot: B392984;
Bcl-2-BX085, Atto 550, Manufacturer: Akoya, PN: 240188, lot: B403537;
TP63-BX093, Alexa Fluor 647, Manufacturer: Akoya, PN: 240179, lot: 1000000302;
b-Catenin1-BX096, Atto 550, Manufacturer: Akoya, PN: 240200, lot: B385075;
Keratin 5-BX101, Alexa Fluor 750, Manufacturer: Akoya, PN: 240189, lot: B389468;
SOX2-BX102, Alexa Fluor 647, Manufacturer: Akoya, PN: 240174, lot: B403163;

Validation

All antibodies used for immunohistochemistry and Multiplexed immunofluorescent histological staining were validated and optimized internally based on the staining conditions and antibody dilution recommended by the manufacturers. Antibodies for flow cytometry were used at dilutions according to manufacturers' recommendation. Antibodies for CODEX and FUSION were validated and optimized internally based on the staining conditions and antibody dilution recommended by the manufacturers.

Eukaryotic cell lines

Policy information about cell lines and Sex and Gender in Research

Cell line source(s)

Six primary cell lines (511950, 60400, 70301, 60531, 511892, 60590) derived from spontaneous PDAC of six different Ptf1awt/Cre;Kraswt/LSL-G12D;Trp53loxP/loxP (CKP) mice, one primary cell line (110299) from spontaneous PDAC of Ptf1awt/Cre;Kraswt/LSL-G12D;Trp53loxPl/R172H (KPC) mouse, and four primary (GP82, GP2838c3, GP99, GP58) LCMV-gp33-expressing cell lines derived from FKPC2GP mice were used. The details of cell lines have been previously reported in Godfrey LK et al, Clin Epigenetics, 2024 and Cheung PF et al, Nat Comm, 2022. 2838c3 and 6694C2 KPCY cell lines were purchased from Kerafast (CAT#EUP013-FP,Boston, USA). The long term trametinib-treated murine PDAC cell lines were established by treatment with increasing doses of trametinib (GSK1120212). GATA6 knockout lines and the derived clones were established using CRISPR-Cas9 in the GATA6high cell line 2838c3. 110299AID-GATA6 cells were generated using the murine primary

	PDAC cells 110299, with the auxin-inducible degron (AID) knock-in approach, as it is thoroughly described in the Materials and Methods paragraph.
Authentication	No authentication was performed.
Mycoplasma contamination	The cell lines were routinely checked for mycoplasma contamination every 2 month. All tests were negative.
Commonly misidentified lines (See ICLAC register)	No commonly misidentified cell lines was used.

Palaeontology and Archaeology

Specimen provenance	*Provide provenance information for specimens and describe permits that were obtained for the work (including the name of the issuing authority, the date of issue, and any identifying information). Permits should encompass collection and, where applicable, export.*
Specimen deposition	*Indicate where the specimens have been deposited to permit free access by other researchers.*
Dating methods	*If new dates are provided, describe how they were obtained (e.g. collection, storage, sample pretreatment and measurement), where they were obtained (i.e. lab name), the calibration program and the protocol for quality assurance OR state that no new dates are provided.*

☐ Tick this box to confirm that the raw and calibrated dates are available in the paper or in Supplementary Information.

Ethics oversight	*Identify the organization(s) that approved or provided guidance on the study protocol, OR state that no ethical approval or guidance was required and explain why not.*

Note that full information on the approval of the study protocol must also be provided in the manuscript.

Animals and other research organisms

Policy information about studies involving animals; ARRIVE guidelines recommended for reporting animal research, and Sex and Gender in Research

Laboratory animals	Ptf1awt/Cre;Kraswt/LSL-G12D; Trp53tm1.1Dgk (CKP) mice, both male and female, 6 weeks old, were used in the study. FKPC2GP mice were generated by crossing Ptf1awt/Flp;Kraswt/FSF-G12D;Trp53tm1.1Dgk (FKP) mice to Gt(ROSA)26Sortm3(CAG-Cre/ERT2)Dsa(R26FSF-CAG−CreERT2) and Gt(ROSA) 26SortmloxP-STOP-loxP-GP-IRES-YFP(R26LSL-GP) strains. For the orthotopic PDAC tumors in mouse, C57Bl/6J (B6J) were used. For the patient-derived xenografts, NOD scid gamma mice were used. Spleens of P14-TCR-Tg mice were used for T-cell isolation.<br>All experimental animals were numbered, genotypes were revealed and animals then assigned to groups for analysis. For treatment experiments mice were randomized. None of the mice with the appropriate genotype were excluded from this study. Details of original and interbred mouse strains were described in the Materials and Methods section.
Wild animals	No wild animals were used in the study.
Reporting on sex	The study was not applied to only one gender. Equal number of female and male mice were used.
Field-collected samples	No field-collected sample was used in the study.
Ethics oversight	The experimentation of animals was authorized by the Landesamt für Natur, Umwelt und Verbraucherschutz Nordrhein-Westfalen under license number 84-02.04.2017.A315.

Note that full information on the approval of the study protocol must also be provided in the manuscript.

Clinical data

Policy information about clinical studies
All manuscripts should comply with the ICMJE guidelines for publication of clinical research and a completed CONSORT checklist must be included with all submissions.

Clinical trial registration	*Provide the trial registration number from ClinicalTrials.gov or an equivalent agency.*
Study protocol	*Note where the full trial protocol can be accessed OR if not available, explain why.*
Data collection	*Describe the settings and locales of data collection, noting the time periods of recruitment and data collection.*
Outcomes	*Describe how you pre-defined primary and secondary outcome measures and how you assessed these measures.*

Dual use research of concern

Policy information about dual use research of concern

Hazards

Could the accidental, deliberate or reckless misuse of agents or technologies generated in the work, or the application of information presented in the manuscript, pose a threat to:

No	Yes	
☒	☐	Public health
☒	☐	National security
☒	☐	Crops and/or livestock
☒	☐	Ecosystems
☒	☐	Any other significant area

Experiments of concern

Does the work involve any of these experiments of concern:

No	Yes	
☒	☐	Demonstrate how to render a vaccine ineffective
☒	☐	Confer resistance to therapeutically useful antibiotics or antiviral agents
☒	☐	Enhance the virulence of a pathogen or render a nonpathogen virulent
☒	☐	Increase transmissibility of a pathogen
☒	☐	Alter the host range of a pathogen
☒	☐	Enable evasion of diagnostic/detection modalities
☒	☐	Enable the weaponization of a biological agent or toxin
☒	☐	Any other potentially harmful combination of experiments and agents

Plants

Seed stocks	*Report on the source of all seed stocks or other plant material used. If applicable, state the seed stock centre and catalogue number. If plant specimens were collected from the field, describe the collection location, date and sampling procedures.*
Novel plant genotypes	*Describe the methods by which all novel plant genotypes were produced. This includes those generated by transgenic approaches, gene editing, chemical/radiation-based mutagenesis and hybridization. For transgenic lines, describe the transformation method, the number of independent lines analyzed and the generation upon which experiments were performed. For gene-edited lines, describe the editor used, the endogenous sequence targeted for editing, the targeting guide RNA sequence (if applicable) and how the editor was applied.*
Authentication	*Describe any authentication procedures for each seed stock used or novel genotype generated. Describe any experiments used to assess the effect of a mutation and, where applicable, how potential secondary effects (e.g. second site T-DNA insertions, mosiacism, off-target gene editing) were examined.*

ChIP-seq

Data deposition

☐ Confirm that both raw and final processed data have been deposited in a public database such as GEO.

☐ Confirm that you have deposited or provided access to graph files (e.g. BED files) for the called peaks.

Data access links<br>*May remain private before publication.*	*For "Initial submission" or "Revised version" documents, provide reviewer access links. For your "Final submission" document, provide a link to the deposited data.*
Files in database submission	*Provide a list of all files available in the database submission.*
Genome browser session<br>(e.g. UCSC)	*Provide a link to an anonymized genome browser session for "Initial submission" and "Revised version" documents only, to enable peer review. Write "no longer applicable" for "Final submission" documents.*

Methodology

Replicates	*Describe the experimental replicates, specifying number, type and replicate agreement.*
Sequencing depth	*Describe the sequencing depth for each experiment, providing the total number of reads, uniquely mapped reads, length of reads and whether they were paired- or single-end.*
Antibodies	*Describe the antibodies used for the ChIP-seq experiments; as applicable, provide supplier name, catalog number, clone name, and lot number.*
Peak calling parameters	*Specify the command line program and parameters used for read mapping and peak calling, including the ChIP, control and index files used.*
Data quality	*Describe the methods used to ensure data quality in full detail, including how many peaks are at FDR 5% and above 5-fold enrichment.*
Software	*Describe the software used to collect and analyze the ChIP-seq data. For custom code that has been deposited into a community repository, provide accession details.*

Flow Cytometry

Plots

Confirm that:

- [ ] The axis labels state the marker and fluorochrome used (e.g. CD4-FITC).
- [ ] The axis scales are clearly visible. Include numbers along axes only for bottom left plot of group (a 'group' is an analysis of identical markers).
- [ ] All plots are contour plots with outliers or pseudocolor plots.
- [x] A numerical value for number of cells or percentage (with statistics) is provided.

Methodology

Sample preparation	For tumor cell surface MHCI and T-cell surface PD1 expression, cells were washed with washing buffer (2% FBS in PBS) and then incubated with primary antibodies or an equal amount of corresponding isotype control.<br>For intracellular tumor GATA6 and Granzyme B, and TNF-a in T-cells, cells were fixed with 4% paraformaldehyde for 10 min at 37°C. After washing twice with PBS, cells were permeabilized with 0.1% Saponin for 20 min and then stained with antibodies and corresponding isotype. Details of primary antibodies are listed in supplementary table. Cells were then washed, resuspended, and subjected to analysis.
Instrument	FACSCelasta, BD Biosciences
Software	FACSDiva, FlowJo version 5.0; BD Biosciences
Cell population abundance	Expression of corresponding molecules of 10,000 viable cells was analyzed by flow cytometry (FACSCelasta; BD Biosciences) as mean fluorescence intensity.
Gating strategy	The FSC/SSC gating strategy was used to exclude cell debris and doublets.

- [ ] Tick this box to confirm that a figure exemplifying the gating strategy is provided in the Supplementary Information.

Magnetic resonance imaging

Experimental design

Design type	*Indicate task or resting state; event-related or block design.*
Design specifications	*Specify the number of blocks, trials or experimental units per session and/or subject, and specify the length of each trial or block (if trials are blocked) and interval between trials.*
Behavioral performance measures	*State number and/or type of variables recorded (e.g. correct button press, response time) and what statistics were used to establish that the subjects were performing the task as expected (e.g. mean, range, and/or standard deviation across subjects).*

Acquisition

Imaging type(s) — *Specify: functional, structural, diffusion, perfusion.*

Field strength — *Specify in Tesla*

Sequence & imaging parameters — *Specify the pulse sequence type (gradient echo, spin echo, etc.), imaging type (EPI, spiral, etc.), field of view, matrix size, slice thickness, orientation and TE/TR/flip angle.*

Area of acquisition — *State whether a whole brain scan was used OR define the area of acquisition, describing how the region was determined.*

Diffusion MRI ☐ Used ☐ Not used

Preprocessing

Preprocessing software — *Provide detail on software version and revision number and on specific parameters (model/functions, brain extraction, segmentation, smoothing kernel size, etc.).*

Normalization — *If data were normalized/standardized, describe the approach(es): specify linear or non-linear and define image types used for transformation OR indicate that data were not normalized and explain rationale for lack of normalization.*

Normalization template — *Describe the template used for normalization/transformation, specifying subject space or group standardized space (e.g. original Talairach, MNI305, ICBM152) OR indicate that the data were not normalized.*

Noise and artifact removal — *Describe your procedure(s) for artifact and structured noise removal, specifying motion parameters, tissue signals and physiological signals (heart rate, respiration).*

Volume censoring — *Define your software and/or method and criteria for volume censoring, and state the extent of such censoring.*

Statistical modeling & inference

Model type and settings — *Specify type (mass univariate, multivariate, RSA, predictive, etc.) and describe essential details of the model at the first and second levels (e.g. fixed, random or mixed effects; drift or auto-correlation).*

Effect(s) tested — *Define precise effect in terms of the task or stimulus conditions instead of psychological concepts and indicate whether ANOVA or factorial designs were used.*

Specify type of analysis: ☐ Whole brain ☐ ROI-based ☐ Both

Statistic type for inference (See Eklund et al. 2016) — *Specify voxel-wise or cluster-wise and report all relevant parameters for cluster-wise methods.*

Correction — *Describe the type of correction and how it is obtained for multiple comparisons (e.g. FWE, FDR, permutation or Monte Carlo).*

Models & analysis

n/a	Involved in the study
☐	☐ Functional and/or effective connectivity
☐	☐ Graph analysis
☐	☐ Multivariate modeling or predictive analysis

Functional and/or effective connectivity — *Report the measures of dependence used and the model details (e.g. Pearson correlation, partial correlation, mutual information).*

Graph analysis — *Report the dependent variable and connectivity measure, specifying weighted graph or binarized graph, subject- or group-level, and the global and/or node summaries used (e.g. clustering coefficient, efficiency, etc.).*

Multivariate modeling and predictive analysis — *Specify independent variables, features extraction and dimension reduction, model, training and evaluation metrics.*
